# Supplementary figures and images for: Matrix vesicles promote bone repair after a femoral bone defect in mice
Source: PLoS One. 2023 Apr 7;18(4):e0284258. doi: 10.1371/journal.pone.0284258 (PMC10081784; doi:10.1371/journal.pone.0284258)

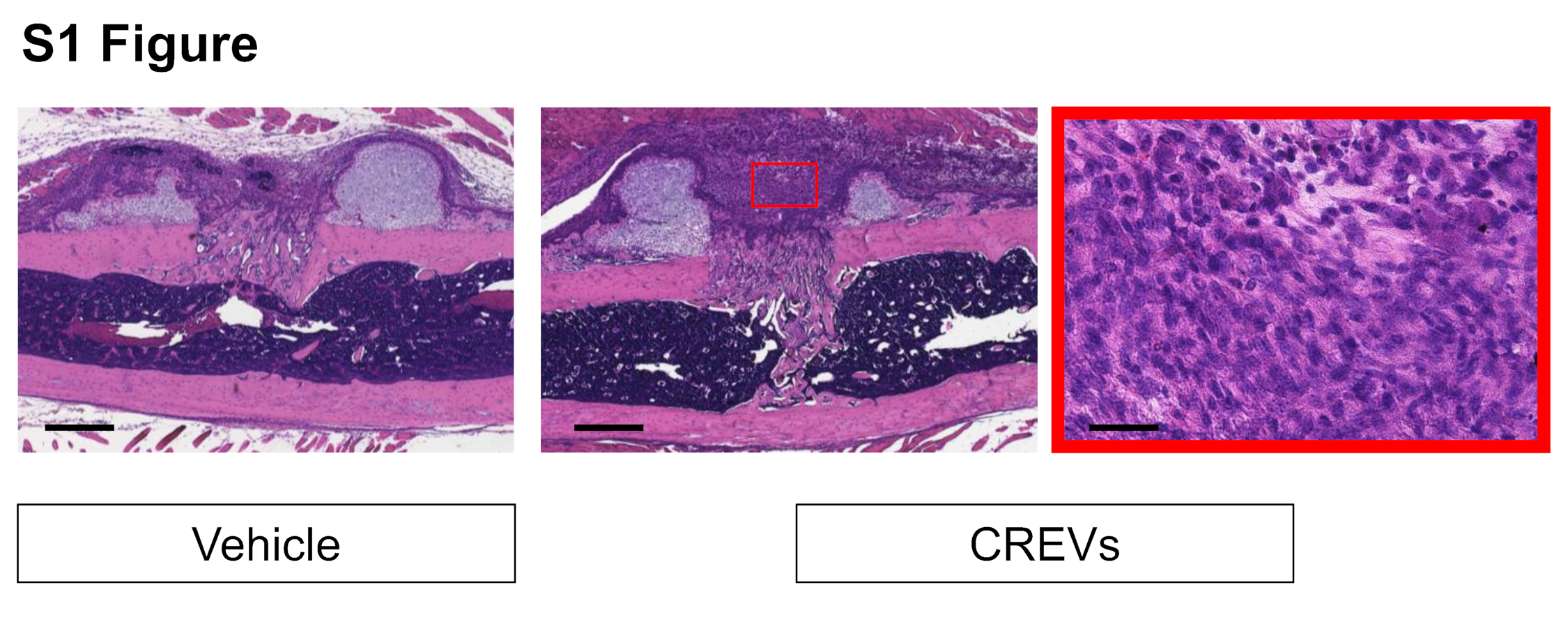

Supplement: S1 Fig — An expanded image of red square region in the center image was presented at right panel. Scale bars in left, center, and right images indicate 500, 500, and 50 μm, respectively. (TIF) [file pone.0284258.s001.tif]

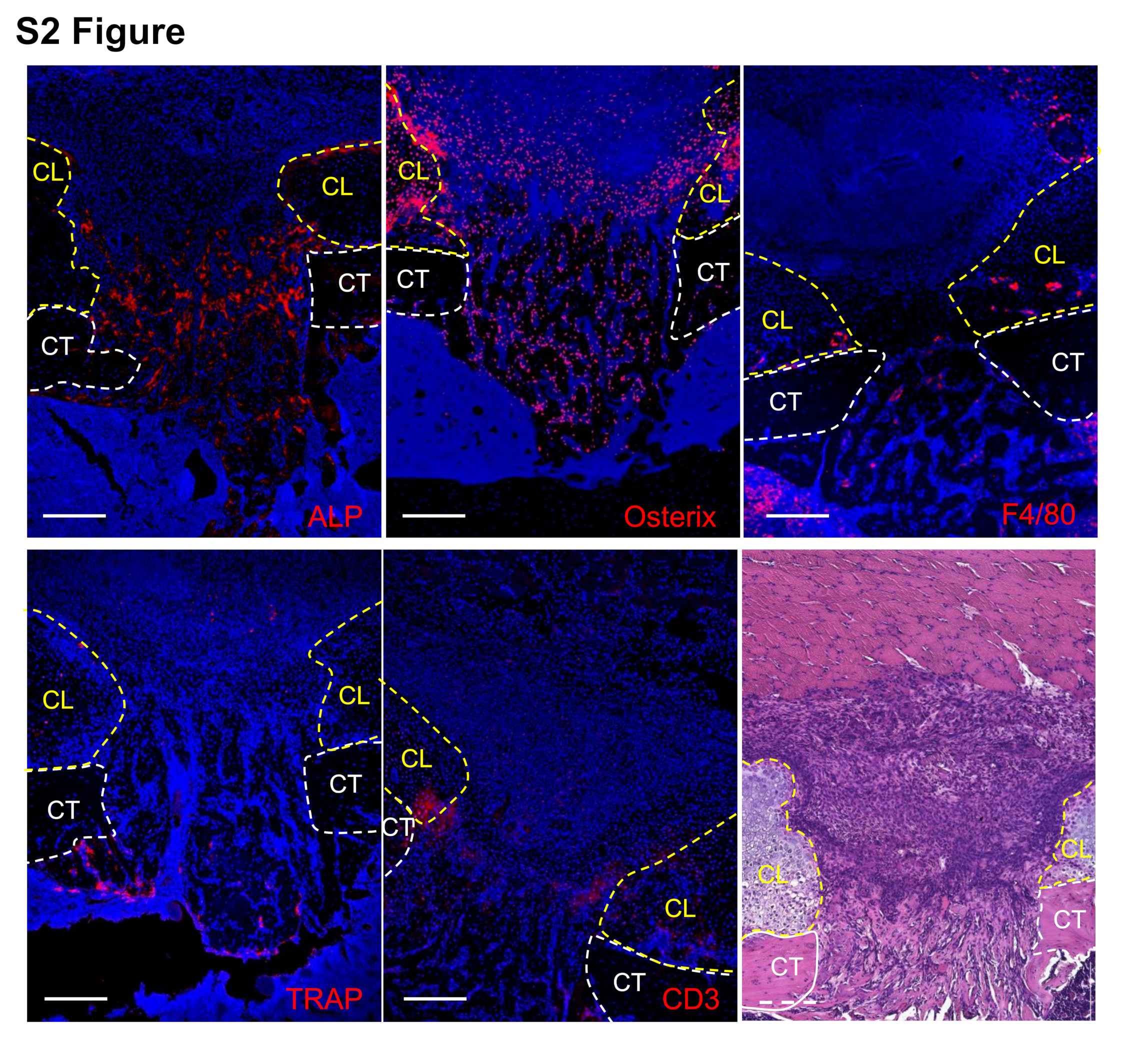

Supplement: S2 Fig — Scale bars indicate 200 μm. The white and yellow line indicates the boundary with cortical bone (CT) and cartilage (CL), respectively. (TIF) [file pone.0284258.s002.tif]

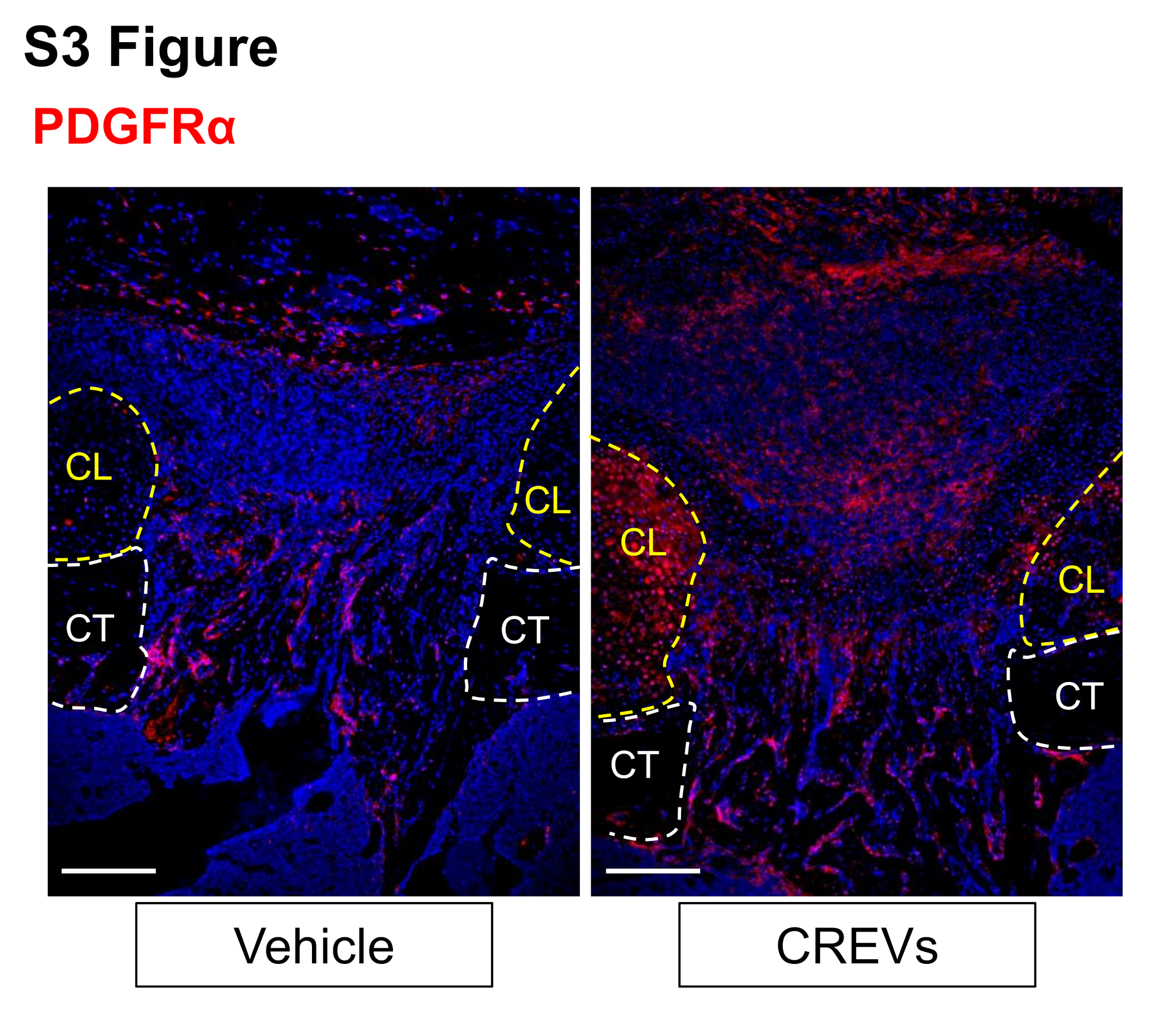

Supplement: S3 Fig — Scale bars indicate 200 μm. The white and yellow line indicates the boundary with cortical bone (CT) and cartilage (CL), respectively. (TIF) [file pone.0284258.s003.tif]

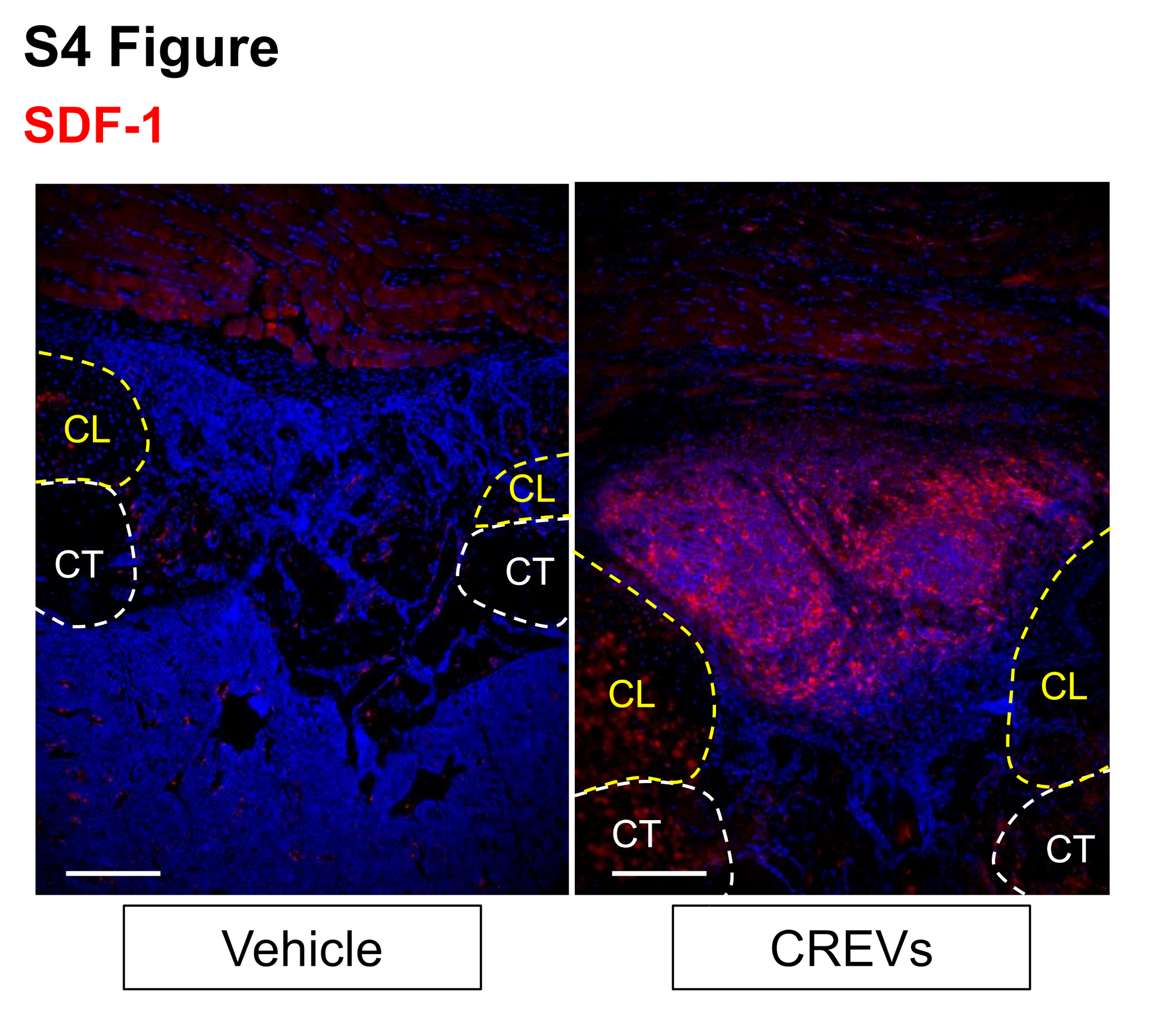

Supplement: S4 Fig — The white and yellow line indicates the boundary with cortical bone (CT) and cartilage (CL), respectively. (TIF) [file pone.0284258.s004.tif]

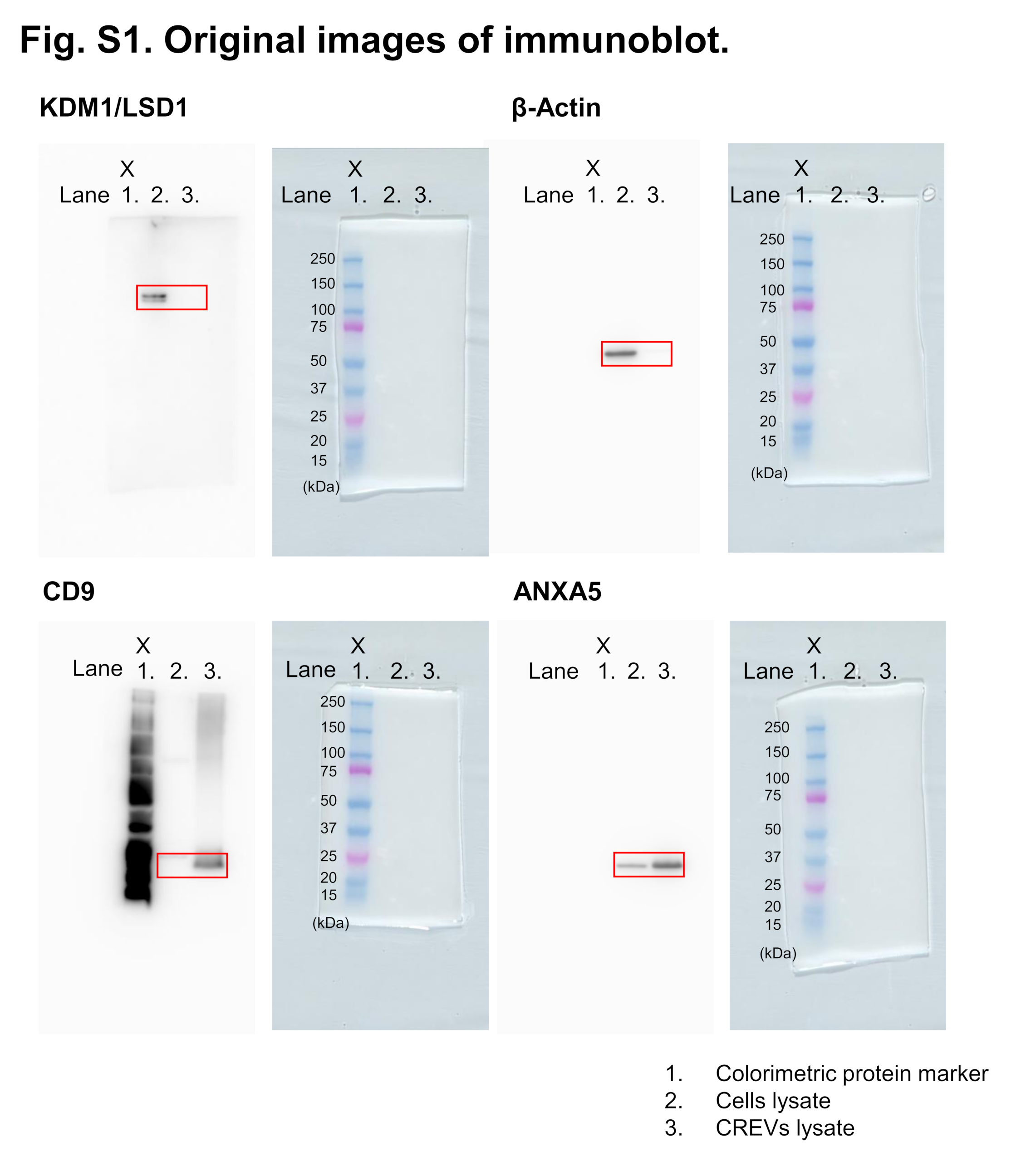

Supplement: S1 Raw images — (TIF) [file pone.0284258.s005.tif]
