# Supplementary material for: Matrix vesicles promote bone repair after a femoral bone defect in mice
Source: PLoS One. 2023 Apr 7;18(4):e0284258. doi: 10.1371/journal.pone.0284258 (PMC10081784; doi:10.1371/journal.pone.0284258)
Supplement: Attachment — Submitted filename: PONE-D-22-31340.docx [file pone.0284258.s006.docx]

PONE-D-22-31340

The authors present an interesting article characterizing collagenase-digested EVs and the use of these in a femoral defect model and the impact of these on osteogenesis *in vitro*. Some claims of the EV identification are debatable, some methodology is missing/unclear, and *in vitro* assessments should more closely match *in vivo* application of CREVs in a hydrogel. The following major revisions are requested:

Introduction:

1. The introduction is lacking detail about the specific bone defect the authors are addressing. The authors claim that a bone defect is generally repaired without clinical intervention and later mention that clinical intervention is needed in some instances of critical-sized defect repair. Authors should re-structure their introduction to bone repair with the focus of femoral defect repair, such as current methods and needs for new approaches, and limitations of current approaches for this specific repair problem.

Methods:

2. Collagenase usage is listed as Units/mL. Define these arbitrary ‘units’.

3. ALP activity is missing methods. Based on figures, it seems this assay was performed on the CREV. Add details explaining what samples were used and how much of these samples were used in this assay.

4. Authors utilize a gelatin hydrogel to deliver CREV to the bone defect site; however, there is no methodology on these hydrogels. Authors need to include information on methods of gelatin hydrogel production, characteristics of hydrogels (porosity, mechanics, etc.), and release characteristics of encapsulated EVs. If this is already published work, then this should be added to the introduction explaining these findings.

5. What was the dosing of CREV used in the mineralization assay? Add this to the methods section.

6. Cell isolation- how were osteoblast cells verified for correct cell isolation? Information on cell line manufacturer for ST2 cells is also needed.

Discussion

7. Authors seem to be claiming that CREVs may be secreted in an exosome-like fashion, but in order to claim this, authors must provide more supporting information than CD9+ and similar size range. This sentence could be removed instead.

8. Authors use CREVs and MtEVs interchangeably but note that CREVs may contain exosome-like EVs and MtEVs, but later claim MtEVs induce bone repair based on CREV results. Authors should clarify the identification of their EVs (likely MtEVs) and use this consistently within the manuscript. For clarity, authors should consider only using MtEVs as an acronym for their EV used in this study.

9. Authors assess osteogenesis in a 2D model *in vitro* but to more accurately represent *in vivo* work, authors should examine the response of osteogenic cells seeded on hydrogels containing CREVs.

10. There was a lack of mineralization differences *in vitro* and authors suggest CREVs could work through a different mechanism. Authors should consider examining cell activity in response to CREVs and protein release of those related to osteoclastogenesis and inflammation (OPG, IL6, etc.), especially from mesenchymal stem cells, as this may prove interesting mechanistic results of CREVs.
