# Supplementary material for: Matrix vesicles promote bone repair after a femoral bone defect in mice
Source: PLoS One. 2023 Apr 7;18(4):e0284258. doi: 10.1371/journal.pone.0284258 (PMC10081784; doi:10.1371/journal.pone.0284258)
Supplement: Attachment — Submitted filename: Response_to_Reviewers.docx [file pone.0284258.s008.docx]

**Responses to the comments of Reviewer #1**

*Re: “The authors address some of the comments within their revised manuscript, but there is still a disconnect between the in vivo observations and the in vitro work done to explain those data. There periosteal reaction with the CREV treatment in enormously different, but that is not explored whatsoever.”*

**(Response)**

We appreciated to the Reviewer’s important remarks on our study. We performed some additional experiments to evaluate the periosteal reaction with the CREV treatment and clarify the mechanisms by which CREVs transplantation enhances chondrogenesis, osteoblastogenesis and bone repair after the femoral bone defect in mice in contrast to the *in vitro* data. Unfortunately, we could not show the vehicle group for Fig. S2 for the lack of residual tissue sections for immunohistochemistry, although they were available for only the evaluation of PDGFRα- and SDF-1-positive cells, which were most important for the additional experiments. Area of larger number of cell accumulation was observed at periosteum site 7 days after the femoral bone defect with CREVs transplantation, compared to vehicle group (Fig. 2, S1). Numerous PDGFRα-positive cells (skeletal stem/progenitor cells (SSPCs)) and Osterix-positive cells (preosteoblastic cells) were observed at periosteum area 7 days after the femoral bone defect with CREVs transplantation (Fig. S2, S3), although few ALP-positive cells (osteoblastic cells), F4/80-positive cells (macrophages), TRAP-positive cells (osteoclasts) and CD3-positive cells (T-lymphocytes) were detected (Fig. S2). In addition, the number of SDF1-positive cells seemed to be increased at the periosteum area by the local administration of CREVs 7 days after the femoral bone defect (Fig S4). These sentences were added to Results (page 23, lines 364-374), and new supplemental Figures (Figure S1-S4) were added.

The following Discussion (page 30, lines 479-495) and 8 references [44-51] were added to assess some mechanisms by which CREVs transplantation enhances chondrogenesis, osteoblastogenesis and bone repair after the femoral bone defect in mice in contrast to the *in vitro* data, “SSPCs are cells with the characteristics of mesenchymal stem cells as well as mobilized from bone marrow, periosteum and skeletal muscles during bone regeneration [44]. SSPCs possess different regenerative potential according to their origin. Namely, SSPCs from bone marrow, periosteum and muscles contribute to osteoblastic bone formation, osteochondral bone formation and cartilage formation, respectively [45-47]. Since the local administration of CREVs seemed to increase numbers of PDGFRα- and Osterix-positive cells as well as cartilage/bone formation at the bone damaged sites in our study, CREVs might recruit SSPCs from bone marrow, periosteum and skeletal muscles, then improving bone regeneration *in vivo*. Our data showed that numerous SDF1-positive cells were observed at the periosteal area of the damaged site with the local administration of CREVs. SDF1 is a chemokine involved in the recruitment of mesenchymal stem cells at the fracture sites [48-51]. Taken together, the local administration of CREVs might recruit PDGFα-positive SSPCs at the bone damaged sites partly through SDF1, leading to an enhancement of chondrogenic/osteoblastic bone formation and subsequent bone repair in mice. Alternatively, MtVs may enhance bone repair through the supply of hydroxyapatite for mineralization. Further studies are needed to clarify this issue.”

**Reference**

44. Julien A, Perrin S, Martínez-Sarrà E, Kanagalingam A, Carvalho C, Luka M, Ménager M, Colnot C, Skeletal Stem/Progenitor Cells in Periosteum and Skeletal Muscle Share a Common Molecular Response to Bone Injury. J Bone Miner Res. 2022 Aug;37(8):1545-1561.
45. Méndez-Ferrer S, Michurina TV, Ferraro F, Mazloom AR, Macarthur BD, Lira SA, Scadden DT, Ma'ayan A, Enikolopov GN, Frenette PS, Mesenchymal and haematopoietic stem cells form a unique bone marrow niche. Nature. 2010 Aug 12;466(7308):829-34.
46. Duchamp de Lageneste O, Julien A, Abou-Khalil R, Frangi G, Carvalho C, Cagnard N, Cordier C, Conway SJ, Colnot C, Periosteum contains skeletal stem cells with high bone regenerative potential controlled by Periostin. Nat Commun. 2018 Feb 22;9(1):773.
47. Julien A, Kanagalingam A, Martínez-Sarrà E, Megret J, Luka M, Ménager M, Relaix F, Colnot C, Direct contribution of skeletal muscle mesenchymal progenitors to bone repair. Nat Commun. 2021 May 17;12(1):2860.
48. Herrmann M, Verrier S, Alini M, Strategies to Stimulate Mobilization and Homing of Endogenous Stem and Progenitor Cells for Bone Tissue Repair. Front Bioeng Biotechnol. 2015 Jun 2;3:79.
49. Link DC, Neutrophil homeostasis: a new role for stromal cell-derived factor-1. Immunol Res. 2005;32(1-3):169-78.
50. Okada K, Kawao N, Yano M, Tamura Y, Kurashimo S, Okumoto K, Kojima K, Kaji H, Stromal cell-derived factor-1 mediates changes of bone marrow stem cells during the bone repair process. Am J Physiol Endocrinol Metab. 2016 Jan 1;310(1):E15-23.
51. Dar A, Kollet O, Lapidot T, Mutual, reciprocal SDF-1/CXCR4 interactions between hematopoietic and bone marrow stromal cells regulate human stem cell migration and development in NOD/SCID chimeric mice. Exp Hematol. 2006 Aug;34(8):967-75.

**Responses to the comments of Reviewer #2**

*Re: “The original comment about the gelatin hydrogels was not fully addressed. Authors have partially answered this comment, but must add some greater detail to the fabrication process of these hydrogels in the methods section to ensure reproducibility. Briefly list fabrication process including how CREVs were encapsulated within the hydrogel into the methods section with references on prior use of these hydrogels.”*

**(Response)**

We apologized for the insufficient explanation about the fabrication process of cationized gelatin hydrogels. The paragraph about “Preparation of cationized gelatin hydrogel sheets” in Materials and Methods was corrected to the follow (page 11, lines 179-197), “Cationized gelatins were synthesized by converting the carboxyl groups of gelatin with the amino groups, previously described [30]. Briefly, 7.8 g ethylenediamine (EDA) was added into 250 ml of gelatin solution (25 mg/ml) in 0.1 M phosphate-buffered solution (PB, pH = 5.0) at 40 °C. The pH of the solution was adjusted to 5.0 by adding 11 M HCl aqueous solution. 5.35 g ethylenedichloride (EDC) was added into the solution and PB was added into the solution to give the final volume of 500 ml. After stirring at 40 °C for 18 h, the gelatin solution was dialyzed against double-distilled water (DDW) for 3 days at room temperature. The dialyzed solution was freeze-dried to obtain cationized gelatins. To determine the percentage of amino groups introduced into gelatin, the conventional 2,4,6-trinitrobenzene sulfonic acid method was performed [31]. The percentage was 19.9 mole% per the carboxyl groups of gelatins. 1.2 ml of cationized gelatin aqueous solution (5.0 mg/ml) was poured into polytetrafluoroethylene mold (43 mm × 43 mm) and freeze-dried to prepare the cationized gelatin hydrogel sheets. The hydrogel sheets were stabilized by dehydrothermal crosslinking at 160℃ for 72 h with dry oven (AURORA DN-305, Sato Vacuum Inc., Japan). Gelatin hydrogel sheets were punched out to circular disc with the diameter of 1.5 mm for the transplantation. 1.5 µL of CREVs suspension in PBS (6.67 µg/µL) was soaked into disc-shaped gelatin hydrogel.”, and two references [30, 31] were added to References.

**Reference**

30. Murata Y, Jo JI., Tabata Y, Preparation of cationized gelatin nanospheres incorporating molecular beacon to visualize cell apoptosis. Sci Rep. 2018 Oct 4;8(1):14839.
31. Snyder SL, Sobocinski PZ, An improved 2,4,6-trinitrobenzenesulfonic acid method for the determination of amines. 1975 Mar;64(1):284-8.

**Responses to the comments of Reviewer #3**

*Re: The authors have addressed all my comments to my satisfaction. However, if they can address the comments made by other reviewers, it will improve the scientific merit of the manuscript.*

**(Response)**

We appreciated for the Reviewer’s positive comments for our manuscript. We added some new data and revised our manuscript to address the other Reviewers’ comments.
